# The Role of Microbiome, Dietary Supplements, and Probiotics in Autism Spectrum Disorder

**DOI:** 10.3390/ijerph17082647

**Published:** 2020-04-12

**Authors:** Bhagavathi Sundaram Sivamaruthi, Natarajan Suganthy, Periyanaina Kesika, Chaiyavat Chaiyasut

**Affiliations:** 1 Innovation Center for Holistic Health, Nutraceuticals, and Cosmeceuticals, Faculty of Pharmacy, Chiang Mai University, Chiang Mai 50200, Thailand; p.kesika@gmail.com; 2 Department of Nanoscience and Technology, Alagappa University, Karaikudi 630003, India; suganthy.n@gmail.com

**Keywords:** autism spectrum disorder, probiotics, diet, microbiome, cognition

## Abstract

Autism spectrum disorder (ASD) is a serious neurodevelopmental disorder characterized by the impairment of the cognitive function of a child. Studies suggested that the intestinal microbiota has a critical role in the function and regulation of the central nervous system, neuroimmune system and neuroendocrine system. Any adverse changes in the gut–brain axis may cause serious disease. Food preferences and dietary patterns are considered as key in influencing the factors of ASD development. Several recent reviews narrated the importance of dietary composition on controlling or reducing the ASD symptoms. It has been known that the consumption of probiotics confers several health benefits by positive amendment of gut microbiota. The influence of probiotic intervention in children with ASD has also been reported and it has been considered as an alternative and complementary therapeutic supplement for ASD. The present manuscript discusses the role of microbiota and diet in the development of ASD. It also summarizes the recent updates on the influence of dietary supplements and the beneficial effect of probiotics on ASD symptoms. An in-depth literature survey suggested that the maternal diet and lifestyle are greatly associated with the development of ASD and other neurodevelopmental disorders. Mounting evidences have confirmed the alteration in the gut microbial composition in children suffering from ASD. However, the unique profile of microbiome has not yet been fully characterized due to the heterogeneity of patients. The supplementation of probiotics amended the symptoms associated with ASD but the results are inconclusive. The current study recommends further detailed research considering the role of microbiome, diet and probiotics in the development and control of ASD.

## 1. Introduction

Autism spectrum disorder (ASD) is one of the serious neurodevelopmental disorders characterized by the impairment of the interactive and social communication ability of a child and the repetitive specific patterns of activities, behaviors (self-injuring activities), abnormal sensitivity and gastrointestinal (GI) difficulties [1,2]. The incidence of ASD has increased rapidly in the last decade. In spite of several extensive studies, the mechanisms and etiology of ASD have not been clearly explained, but some of the environmental factors such as viral infection, metabolic disparities and the exposure to toxins, diet, genetic and postnatal factors and microbiome have been associated with the etiology of ASD. There are no effective and accorded therapies available for ASD [3,4,5,6,7].

Several recent studies revealed that the intestinal microbiota has a critical role in the function and regulation of the central nervous system, neuroimmune system and neuroendocrine system. Any adverse changes in the gut–brain axis may cause serious diseases such as autoimmune disorders [8,9]. The GI symptoms are associated with several diseases and disorders [10] and about 12% of ASD patients are suffering from GI symptoms [6], which are closely correlated with the unique microbial composition of ASD patients compared to healthy individuals [11].

Food preferences and dietary patterns are also considered as one of the key influencing factors of ASD development. Several recent reviews narrated the importance of dietary composition on controlling or reducing the ASD symptoms [7,12,13]. The quality in terms of nutritional value and quantity of the food significantly alters the microbial composition of the host GI system. The consumption of specific food(s), which is often observed among the ASD patients, affects the microbiota (supports the nourishment of a particular group of microbes).

Several studies proved that the probiotics (a group of live microbes that confers health benefits upon being consuming in an adequate amount) supplementation confers several health benefits by the positive amendment of gut microbiota [14,15,16,17]. The influence of probiotic intervention in ASD patients has also been reported and considered as an alternative and complementary therapeutic supplement for ASD [7,13].

This review preparation was carried out by collecting related scientific information from Scopus, PubMed, Google Scholar and ASD-related databases using the keywords “autism” “autism and probiotics” and “autism and microbiota”. The relevant papers published in English were selected for the preparation of the manuscript without any chronological restrictions. The current manuscript narrates the updated information about the role of microbiome and diet in the development of ASD. This manuscript also explains the beneficial effect of dietary supplements and probiotics in the improvement of ASD symptoms. This review highlights that probiotics can be used as an adjuvant therapeutic agent and with added nutritious support (dietary supplements) but suggests that still more detailed studies are required to develop more efficient therapeutic methods to improve the quality of life of the ASD children.

## 2. Autism Spectrum Disorder 

Autism spectrum disorder (ASD) refers to a group of neurodevelopmental disorders characterized by the impairment of the social interaction and communication skills with rigid and repetitive behaviors. Autism affects both children and adults, based on severity and intellectual ability; they may either lead a normal life or suffer a devastating disability requiring institutional care [18]. Distinctive symptoms of ASD are deficit in social behaviors and nonverbal interactions such as the avoidance of eye contact, inability to control emotion or understand the emotions of others, facial expression and body gestures in the first three years of life. Paul Eugen Bleuler is a swiss psychiatrist that coined the term “autism” (Greek word “autos” means “self”) for group of symptoms related to schizophrenia, while Hans Asperger and Leo Kanner designed the modern study of autism. An epidemiological survey revealed that ASD is the most prevalent non immune mediated CNS disorder with an incidence rate of 1 ASD per 500 children aged eight years, with a higher incidence in boys (23.6 per 1000) when compared to girls (5.3 per 1000) [19]. ASD-affected individuals exhibit unusual ways of learning and reactions to sensation. ASD is a multifactorial disorder caused by the interaction of both genetic and non-genetic risk factors.

### 2.1. Etiological Factors Leading to Autism

Mounting evidences revealed that de novo mutations, copy number variations, rare and common variants are major genetic factors leading to ASD. Around 50% of ASDs are hereditary caused due to defects in the gene and chromosomal abnormalities leading to disruption in the neuronal connection, brain growth and synaptic morphology [20,21,22,23]. Siblings born in families with ASD have a 50% enhanced risk of ASD with a reoccurrence rate of 5%–8%. In monozygotic twins, the concordance rate is 90%, while in dizygotic twins the rate of incidence is 10% [24]. Genetic studies revealed that a mutation in the single gene involved in synaptogenesis alters the developmental pathways of neuronal and axonal structures. The fragile X syndrome, tuberous sclerosis, hyper excitability of neocortical circuits and abnormal neural synchronization were considered as probable disorders leading to ASD [25,26]. In-depth genomic studies revealed that the chromosomes 2q, 7q, 15q and 16p have genes susceptible for ASD that have not yet been completely studied [27]. Inborn metabolic errors such as phenylketonuria, creatine deficiency syndromes, adenylosuccinate lyase deficiency and metabolic purine disorders account for 5% of ASD incidence. Recent reports revealed that the gene ENGRAILED 2 mainly involved in cerebellar developmental patterning, GABA system genes and serotonin transporter genes, has been considered to be associated with ASD [28]. 

**Figure 1:**
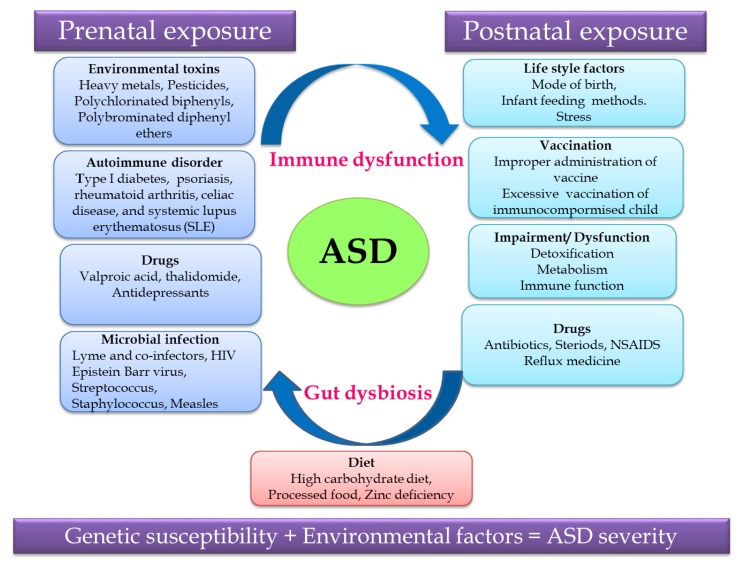
Putative autism spectrum disorder (ASD)-related and environmental factors contributing to ASD.

Non-predisposed factors such as exposure to environmental factors and pharmaceutical drugs, autoimmune disorder, microbial infection and diet during the prenatal and postnatal periods cause gut dysbiosis and immune dysregulation together contributing to ASD (Figure 1). Recent research revealed that the severity of ASD depends on the complex interaction of genetic susceptibility and environmental factors, so unraveling this relationship will help in identifying a treatment strategy for ASD [29,30]. 

### 2.2. Link between Gut Microbiome and ASD

#### 2.2.1. Role of Gut Microbiota in Human Nutrition and Health

Gut–brain cross talk involves a complex communication system involved in the proper maintenance of GI homeostasis, which is termed as the gut–brain axis (GBA). The GBA is a bidirectional communication network between the central nervous system (CNS) and the enteric nervous system linking the emotional and cognitive centers of the brain with peripheral intestinal functions via neuro–immuno–endocrinal mediators [31]. The bidirectional communication network of the GBA involves the brain and spinal cord of the CNS, autonomous nervous system (ANS), the enteric nervous system (ENS) and hypothalamic pituitary adrenal axis (HPA). Sympathetic and parasympathetic limbs of ANS transmit the afferent signals from the lumen to the CNS through the enteric, spinal and vagal pathways and efferent signals from the CNS to the intestinal wall [32]. The HPA axis is the core stress efferent axis, which adapts the organism to various stresses and is part of the limbic system involved in memory and emotional response. On exposure to stresses such as environmental factors and pro-inflammatory cytokines, the HPA axis activates the release of the corticotrophin release factor (CRF) from the hypothalamus, stimulating the secretion of the adrenocorticotrophic hormone (ACTH) from the pituitary gland, which in turn activates the adrenal gland to release the stress hormone cortisol affecting various organs including the brain. Hence, both neuronal and hormonal interactions play a vital role in influencing the activities of intestinal functional effector cells, such as immune cells, epithelial cells, enteric neurons, smooth muscle cells, interstitial cells of cajal and enterochromaffin cells which in turn are under the control of gut microbiota via brain–gut reciprocal communication [33]. Moreover, the epithelial cell lining of the GI tract and its motility, which is controlled by the CNS, influences the composition of gut microbiome. Hence, any dysregulation in the CNS alters the intestinal microbiota leading to pathological consequences and gut microbial dysbiosis which affects the development and regulation of the hypothalamic–pituitary–adrenal axis (HPA) and behavior. This bidirectional relationship of gut microbiome with the host brain axis is termed as microbial endocrinology or inter-kingdom signaling [34].

Gut microbiota interacts with the brain through endocrine and neurocrine pathways, while the brain influences the microbial composition through the autonomic nervous system with the active involvement of the immune and humoral systems. Gut microbiome modulates the brain by influencing the production of neurotransmitters such as serotonin, gamma amino butyric acid (GABA) and the brain-derived neurotrophic factor (BDNF) via short chain fatty acid, tryptophan metabolites, secondary bile acids and ketones thereby influencing memory and learning processes. These molecules transmit signals by interacting with the farnesoid receptor (FXR) and G protein-coupled bile acid receptor (TGR5) in the enteroendocrine cells (EEC), releasing fibroblast growth factor (FGF19), which readily crosses the blood–brain barrier and regulates the secretion of neuropeptide Y in the hypothalamus, thereby regulating the glucose metabolism via the release of glucagon-like peptide (GLP-1) [35,36,37]. The release of serotonin (5 hydroxy tryptamine-5HT) by enterochromaffin cells (ECC), triggered by the stimuli from efferent neurons of the CNS based on the availability of the dietary tryptophan level, which in turn is controlled by gut microbiome, represents the bidirectional gut microbiome–brain axis [38]. Certain microbially-derived molecules escape the intestinal barrier, reach the brain directly by crossing the blood–brain barrier via systemic circulation and propagate the signal on interaction with the FXR and TGR5 expressed in brain neurons [39]. 

**Figure 2:**
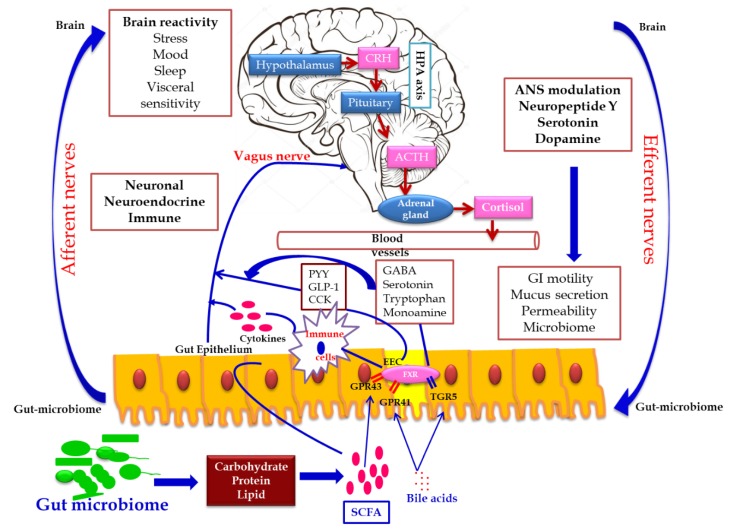
Gut microbiome–brain axis: bidirectional signaling pathways illustrating the relationship between the gut microbiome, intestinal barrier and the brain. Gut microbiota communicates with the brain through the neuro–endocrine–immune network either indirectly via the gut-derived molecules acting on afferent vagal nerve endings, or directly via the microbe-generated signals. The brain’s structural connections (the multiple interconnected structural networks of the central nervous system) regulates the gut microbiota via the autonomic nervous system. Disturbance in the bidirectional interaction response gain is due to psychosocial or gut-derived stress manifests to brain–gut disorders.

In addition, the secretion of biologically active peptide by enteroendocrine cells, mainly involved in GBA interaction, is controlled by the nutritional level of microbiota. SCFA acts as the major signaling molecule mediating the gut microbiome–brain communication, via EEC and ECC. The brain influences the microbial population through several stresses: by altering the size and quality of the mucus secretion, by slowing the recovery of the migratory motor complex pattern, by the induction a transient delaying of gastric emptying, and by enhancing the frequency of cecocolonic spike burst activity, which affect the GI transit modulating the nutrient supply to enteric microbiota. Different psychological stressors in adults and newborns modulate the composition and biomass of enteric microbiota [40] (Figure 2).

#### 2.2.2. Link between Gut Microbiome and ASD

Gut microbiota, which is non-genetic and inheritable, has a great impact on immune, metabolic and neuronal developments. As gut microbiota is a notable contributor for human health, gut microbial dysbiosis leads to negative consequences such as GI-tract-related disorders such as Crohn’s disease and ulcerative colitis, systemic diseases such as metabolomic disorders and CNS-related disorders [41]. Noticeable evidences illustrated that ASD patients in addition to psychiatric disorders were found to be associated with an extremely painful GI disease termed as autistic enterocolitis or other GI discomforts such as constipation, diarrhea and bloating. This hypothesis leads to fact that microbial imbalance affects the co-ordination of the microbiota-gut–brain axis in human health leading to several neurological disorders which turned the focus of the researcher towards gut microbiota. 

Wakefield and colleagues reported the incidence of a new variant inflammatory bowel disease (autistic enterocolitis), which is characterized by chronic patchy inflammation and lymph nodular hyperplasia in the ileum or colon of individuals with ASD [42]. Scientific evidences also illustrated the relationship between ASD patients and gut microbiome, which has a direct/indirect influence over the feeding pattern and nutrition [43]. Investigation has shown that children with autism suffer from intestinal dysbiosis characterized by the imbalance between beneficial microbes and pathogenic microbes residing in the gut. Neurotoxic and cytotoxic molecules, such as opioid peptides, produced by these pathogenic bacteria enter the blood stream due to leaky gut, thereby activating the immune mechanism causing tissue damage and GI inflammation. In addition, these toxic molecules affect the neurotransmitter function in the brain, leading to abnormalities in behavioral patterns such as decreased socialization, decreased response to pain, abnormal language and self-abusive or repetitive behaviors, resulting in confusion, delirium and even coma [32]. In ASD-vulnerable children, yeast also produces abundant chemicals leading to neurological effects. 

The metagenomic analysis of ASD-gut-microbiome showed mucosal dysbiosis with a low level of *Bacteriodetes* and an increased ratio of *Firmicutes* to *Bacteriodetes*. Pyrosequencing of fetal microflora in the fecal stools of autistic children showed a high prevalence of *Desulfovibrio* species and *Bacteroides vulgatus* when compared to healthy volunteers [44]. The 16S r DNA sequencing of gut microbiome, isolated from late onset autism patients, showed a high incidence of *Clostridium* and *Ruminococcus* species, while a real-time PCR analysis showed a rich source of *Clostridium* cluster groups I and XI and *Clostridium bolteae* [45]. Culture-independent fluorescence in situ hybridization studies revealed the elevated level of *Clostridium hystolyticum* in the ASD children compared to healthy children [46]. The accumulation of neurotoxin-producing bacteria such as Clostridia worsens the autistic symptom. Another study on the gut microbial composition of autistic children revealed a low level of *Bifidobacterium* and *Enterococcus* and an increased level of *Lactobacillus* strains, despite being beneficial, which is quite paradoxical. Commensal bacteria such as *Bacillus* spp. and *Klebsiella oxytoca*, that are neither harmful nor beneficial, were reported to be present in autistic children [47]. A pilot study by Kang et al. [48] reported the presence of low-level carbohydrate degrading/fermenting bacteria such as *Prevotella, Coprococcus* and *Veillonellaceae* in ASD, substantiating the link between gut microbiome and ASD. Pyrosequencing results showed the altered gut microbial diversity in autistic children with a relatively high abundance of *Caloramator*, *Sarcina* and *Clostridium* genera, *Alistipes* and *Akkermansia* species, *Sutterellaceae* and *Enterobacteriaceae* and low level of *Prevotella*, *Coprococcus* and unclassified *Veillonellaceae*, concomitantly associated with the altered level of free amino acids and volatile organic compounds in fecal samples compared to the control samples [49]. The above reports illustrate altered gut microbiota in patients suffering from autism, when compared to healthy volunteers, which shows the direct/indirect relationship of gut microbiome with autism.

#### 2.2.3. Microbial Metabolites Interrelated with ASD

The metabolites produced by gut microbiome affect the neural process based on their level. Metabolomic studies in urine, fecal and serum samples of ASD patients using LC-MS and GC-MS revealed an enhanced level of microbial metabolites, such as increased levels of SCFAs, para-cresol and ammonia, which affect the neural process [50,51]. SCFA is the double edged sword that plays a vital role in human health and disease. SCFA is considered as a major trigger factor for ASD. Propionic acid, the widely used preservative in the food industry, is also one of the SCFAs produced by ASD-associated bacteria, such as *Clostridium*, *Bacteroides* and *Desulfovibrio.* Experimental studies with rodents treated with propionic acid exhibited ASD-associated symptoms, such as impaired and restricted social behavior and cognition, together with an enhanced neuro-inflammatory response, which might be due to alteration in mitochondrial function or the epigenetic modulation of ASD-associated genes [52]. The elevated level of another microbial metabolite para-cresol (*p*-cresol) and its conjugate *p*-cresylsulfate were observed in the urinary samples of children affected by autism. The increased level of *p*-cresol, derived either from the environment or gut microbiome, aggravates the ASD severity by inhibiting the neurotransmitters associated with enzymes and cofactors required for sulfonation reaction in the liver [53]. 

Urinary metabolomic research in ASD children revealed the presence of an abnormal level of common microbial metabolites such as dimethylamine, hippuric and phenylacetylglutamine and altered tryptophan, when compared to healthy children [54,55]. The increased level of tyrosine analogue 3-(3-hydroxyphenyl)-3-hydroxypropionic acid (HPHPA), reported in the urine samples of autistic patients, might be responsible for the catecholamine depletion, worsening autistic symptoms such as stereotypical behavior, hyperactivity and hyper-reactivity [56]. Serum metabolomic studies depicted the presence of 11 metabolites in abnormal level among which, the level of sphingosine 1-phosphate and docosahexaenoic acid were consistent in all models [57]. The abnormal gut microbiome-associated metabolites in ASD patients revealed an altered metabolism leading to the aberrant increase in metabolites, worsening the symptoms of ASD.

#### 2.2.4. Gut Microbiome-Associated Immune Deregulation 

Several studies in human and animal models of ASD revealed the presence of an enhanced level of pro-inflammatory cytokines, brain-specific auto-antibodies in the cerebrospinal fluid and serum, illustrating an elevated immune response substantiating the fact that immune dysregulation acts as a key factor contributing to the pathophysiology of ASD [58,59]. Further autopsy of the brain specimens of ASD patients showed the presence of activated microglial cells, together with an increased level of cytokine, such as interferon (IFN)-γ, IL-1β, IL-6, IL-12p40, tumor necrosis factor (TNF)-α and chemokine C-C motif ligand (CCL)-2 [60,61]. Elevated cytokines and chemokines were related to aberrant stereotypical behavior and cognitive impairment. In the newborn infants, the immune system development and homeostasis are regulated by the maternal gut microbiome colonizing the fetal gut. Multiple evidences revealed that the immune dysregulation in ASD is associated with gut microbiota [62]. Certain species of gut microbiota regulate the T lymphocyte differentiation, while few microbes, such as *Bacteroides fragilis* colonization, restrict T helper cell response eliciting an autoimmune disorder [63]. Although several studies reported that impairment of the immune system in ASD is linked with gut microbiome, the mechanism behind it is not clearly known.

#### 2.2.5. Maternal Risk Factors Regulating Gut Microbiome

Epidemiological and experimental studies revealed a strong linkage between maternal infection and the development of ASD in the offspring. The gut microbial composition of a newborn infant varies with respect to the mode of delivery primarily colonized by the maternal microbiota. Hence, any imbalance in maternal microbiome with respect to environmental stress or genetic risk will be transferred to the offspring at the time of birth [64,65]. Scientific evidences revealed that a maternal high-fat diet and exposure to stress during the gestation period increases the risk of neurodevelopment and behavioral disorders in offspring [66]. Maternal immune activation studies using animal models exposed to poly (I:C) during the prenatal stage revealed a change in gut microbiome, leading to lifelong neuropathology and altered behaviors in the offspring [67,68,69]. Based on the reports, it is clear that maternal risk factors increase the incidence of ASD in the offspring by multiple pathways, such as the altered modulation of the placenta, epigenetic modification and immune dysregulation.

## 3. Diet and ASD

Studies strongly showed that the maternal diet plays a critical role in the ASD development in their offspring [70,71]. Generally, the consumption of sufficient amounts of folic acids and vitamins during pregnancy are greatly associated with the decreased risk of ASD [72,73]. A maternal high-fat diet is associated with the high risk of neurodevelopmental disorders and ASD [12,66,70]. 

### 3.1. Recommended Food Supplements for ASD Children

Individuals with ASD have a serious problem in their nutrition due to the stringent food selection, defects in food digestion and absorption. Children with ASD show high selectivity towards food containing starch, snacks and processed food, but deny fruits, vegetables and proteins [43]. It is known that most autistic children are underweight due to a lack of dietary fibers, vitamins, calcium, iron and potassium [74]. Studies also revealed that the nutritional supplementation to ASD children that exceeds their daily need of protein, carbohydrates and fats leads to GI problems and obesity [75,76]. 

Ketogenic diets (KD) that are rich in fat (65–90%) are the common choice to manage the ASD. Several studies revealed some of the favorable impacts of KD on the behavior and symptoms of ASD in human and animal models [77,78,79]. However, several issues must be considered before using KD for treating the condition of ASD in human, such as GI-associated conflicts, food selectivity, low tolerance level of the food and nutrient deficiency due to low consumption [80,81]. Other than KD, several nutritional supplements such as omega-3-fatty acids, vitamins, minerals and antioxidants are believed to exhibit favorable effects on ASD. 

Omega-3 fatty acids (ω-3) are generally used as a food supplement and considered as a potent complementary and alternative therapeutic agent for ASD [82]. Omega-3 long-chain polyunsaturated fatty acids (PUFAs), especially docosahexaenoic and eicosapentaenoic acids, are necessary for the normal brain and visual development and function and regulation of behavior and mood [83]. The defective metabolism of PUFAs has been observed in ASD children, which was associated with an increase in inflammatory cytokines, oxidative stress and the malfunctioning of neurotransmitters [84]. A low level of ω-3, altered ω-3 and ω-6 ratio have been detected in ASD children compared to that of the normal children [85]. Clinical studies based on the impact of the supplementation of ω-3 on the health status of ASD children showed no promising beneficial effect and was found to be controversial [86,87,88]. Despite that some of the ASD-associated symptoms were improved by the ω-3 supplementation, further detailed studies with increased sample size and proper follow-up are required to establish strong evidence for the beneficial effect of ω-3 on ASD [89].

Oxidative stress is one of the factors associated with the etiology of ASD and the defect in the antioxidant system may affect the brain function and disturb the immune system [90,91,92]. The supplementation of antioxidants (such as vitamins, flavonoids) ameliorates symptoms of ASD [93,94], but the evidence is not enough to recommend an antioxidant-based therapeutic practice for ASD.

Several studies have been reported based on the role of the supplementation of minerals and multi-vitamins in improving the symptoms of ASD [95,96,97]. However, additional studies are required [98] to develop minerals and vitamins-based supplementation for the betterment of the symptoms of ASD.

Other food supplements such as fermentable saccharides and a polyols diet are also believed to have a protective effect on ASD. However, scientific evidence is lacking to support the aforementioned statement. Due to calcium deficiency, ASD children suffer from defective intestinal permeability, which roots for several GI-associated diseases and discomforts. In addition, the evidence to support the beneficial effects of the Feingold diet (without any artificial flavors, colors and food additives) is not sufficient. The results of the previous studies claimed that further extended research is essential before recommending any kind of food-based alternative therapy for ASD [7].

### 3.2. Elimination Foods of ASD

The exclusion of some of the food materials is also significantly considered to improve the ASD and/or to prevent the worsening of the ASD. A gluten-free and casein-free (GFCF) diet is considered as one of the effective food-based alternative treatments for ASD since the peptides derived from gluten and casein can trigger inflammation [99,100]. Although studies claimed that the GFCF diet has a positive impact on the health status of ASD patients [101,102], the results are questionable due to the lack of proper methodology, follow-ups, small sample size and most of the data being collected from the parents of ASD children. Some of the studies showed that a GFCF diet does not influence ASD symptoms [103,104]. Hyman et al. [105] reported that specific dietary supplementation had no significant effect on the symptoms of ASD, whereas a randomized controlled study conducted by Ghalichi et al. [106] suggested that a gluten-free diet had a positive impact on the GI health and behaviors of ASD children. Apart from health benefits, though the results are inconsistent, GFCF diet plans showed some serious side effects due to the lack of calcium and deficiency in essential amino acids, which may cause reduced bone density and frequent bone fractures [107,108].

Recent evidences showed that a high-fat diet (differed from KD; a complex of high fat, sufficient amount of protein and low carbohydrate food) affects the dopamine system (such as decreasing the signaling and dysfunction) and causes some of the ASD-associated behavioral changes [109,110,111].

## 4. Influence of Probiotic Supplementation on the Health Status of Individuals with ASD

Parracho et al. [112] conducted a placebo controlled, double blind, crossover-designed feeding trial (12 week study duration; one group subjected to three weeks of placebo treatment followed by three weeks of washout period and followed by probiotic treatment for three weeks then three weeks of washout period; another group subjected to three weeks of probiotic treatment followed by three weeks of washout period and then with placebo treatment for three weeks, followed by three weeks of washout period) and studied the beneficial effect of probiotic strain *Lactobacillus plantarum* WCSF1 on the gut microbiota, gut function and the behavior of the children with ASD. Supplementation of *L. plantarum* WCSF1 (4.5 × 10^10^ CFU per day) to the ASD children (4 to 16 years old) significantly altered the fecal microbiota, a notable level of increase was observed in enterococci and lactobacilli group and a reduction was observed in *Clostridium* cluster XIVa. Bowel function was improved during the probiotic intake compared to the placebo feeding. The GI symptoms, such as intestinal bloating, abdominal pain and flatulence, were found not to be significantly altered, but stool consistency was observed to be improved significantly during the probiotic feeding compared to that of the placebo treatment. The behavioral scores assessed using the Development Behavior Checklist questionnaire were found to be improved during the probiotic feeding compared to baseline. The study suggested that the supplementation of WCSF1 amended the microbiota and behavior of ASD children [112].

Tomova et al. [113] investigated the GI microbiota in children with ASD, their siblings and neurotypical children (control) in Slovakia and also studied the changes in the fecal microbiota, plasma hormone and cytokine levels after probiotic intervention in ASD children, their siblings and the control children. Daily supplementation of three *Lactobacillus* strains, two *Bifidobacterium* strains and a *Streptococcus* strain (mixed in a 60:25:15 ratio; one capsule thrice a day) for four months decreased the amount of *Bifidobacterium* and *Desulfovibrio* spp. and also normalized the ratio of Bacteroidetes/Firmicutes in the feces of ASD-children. The study showed that the severity of the ASD has a strong positive correlation with the severity of the GI dysfunctions in the subjects. The level of TNFα was decreased after probiotic supplementation. No strong correlations were found between the plasma levels (DHEA-S, oxytocin and testosterone) and fecal microbiota. Overall, probiotic supplementation altered the composition of gut microbiota in ASD children [113].

A study by Adams et al. [11] revealed that the severity of ASD has a strong direct correlation with the GI symptoms, which was assessed by the Autism Treatment Evaluation Checklist (ATEC), and that the probiotic supplementation influences the level of short chain fatty acids in ASD children.

d-arabinitol is a metabolite of pathogenic *Candida* spp. and the ratio of d-arabinitol/l-arabinitol is one of the biomarkers of candidiasis. The supplementation of the probiotic (*L. acidophilus* Rosell-11; 5 × 10^9^ CFU per gram; twice a day) for two months to autistic children significantly reduced the level of urine d-arabinitol and d-arabinitol/l-arabinitol ratio and also improved the concentrating power and the ability to respond to an order [114]. 

A case study reported by Grossi et al. [115] revealed that the supplementation of VSL#3 (a mixture of live cells of *Lactobacillus delbrueckii* subsp. *Bulgaricus* (reclassified as *L. helveticus*), *L. acidophilus, B. breve, B. longum, B. infantis*, *L. paracasei, L. plantarum, S. thermophilus*) for four weeks significantly improved the autistic core symptoms and reduced the severity of the GI symptoms in a boy (12-years-old) with ASD and a severe disability of cognitive function. Moreover, during the follow up treatment period (four months), a VSL#3 intervention reduced the Social Affect domain of Autism Diagnostic Observation Schedule (ADOS) score after two months of treatment and further reduced the score after another two months. There was no fluctuation in the ADOS score up to 10 months (follow up period). This study suggested that the proper use of probiotics may improve the ASD symptoms, but more research is necessary [115].

The supplementation of a mixture of probiotic strains (*B. longum*, *L. rhamnosus*, *L. acidophilus*; 1 × 10^8^ CFU per gram; 5 g per day) for three months significantly altered the fecal microbiota of ASD children, specifically the level of Bifidobacteria and Lactobacilli, which were found to be increased after intervention compared to baseline. The severity of the ASD and the severity of the GI symptoms, were measured by ATEC and a six-item GI Severity Index (6-GSI) questionnaire, respectively, and were found to be reduced after probiotic supplementation in ASD children compared to baseline. The study suggested that probiotic supplementation improves the behavioral pattern and the GI discomforts in ASD children [116] (Table 1).

*L. rhamnosus* GG (10^10^ CFU per day) was supplemented to pregnant women for four weeks before delivery and continued the probiotic supplementation for six months to the mother (if breastfeeding) or to the infant. The clinical evaluation and behavioral assessments were made at three weeks and at 3, 6, 12, 18 and 24 months of age and final attention-deficit hyperactivity disorder (ADHD) and Asperger syndrome (AS) records were made at the age of 13. The results showed that probiotic supplementation reduced the risk of the development of a neuropsychiatric disorder compared to that of the placebo group [117].

## 5. Conclusions

Mounting evidences confirmed the alteration in the gut microbial composition in children suffering from ASD. However, the unique profile of microbiome has not yet been fully characterized due to the heterogeneity of patients. GI disorders such as bowel dysfunction and GI tract inflammation are more frequent in severe cases of ASD. The findings of several recent studies showed that amendments of gut microbiota may improve the ASD symptoms and revealed the beneficial impact of probiotic supplementation on the improvement of symptoms of ASD, which suggest that probiotics can be used as a potent adjuvant therapeutic agent for neurodevelopmental disorders such as ASD. In addition, studies have suggested that the dietary regulations may strengthen the therapeutic advancement in ASD treatment. However, sufficient research evidence is missing to authenticate the potential effects of probiotics and dietary interventions on the symptoms of ASD. More scientific research is needed to address the issues such as the optimum dose and duration of probiotic supplementation for ASD treatment, the fate of microbial metabolites in the human body and the compatibility of dietary supplements and probiotics, which would help us to develop typical and effective treatment strategies to improve ASD symptoms. Overall, the improvement of the life quality of ASD children requires mutual and moral support from parents and society, in addition to therapeutic approaches.

## Figures and Tables

**Table 1:** The effect of probiotic supplementation on the health status of individuals with ASD.

Subjects	Probiotics	Dose and Duration	Key Findings	Ref.
ASD-Children (2.5 to 18 years old)	Any type of probiotic	Daily usage (33%)	Low level of short chain fatty acids	[11]
ASD-Children (4 to 16 years old)	*Lactobacillus plantarum* WCSF1	4.5 × 10^10^ CFU per capsule per day for 3 weeks in the 12 week study duration	↑ Enterococci and Lactobacilli group. ↓ Clostridium cluster XIVa Improved the stool consistency compared to placebo, and behavioral scores compared to baseline	[112]
ASD-Children (2 to 9 years old); Their siblings (5 to 7 years old); Chidren in control group (2 to 11 years old)	3 *Lactobacillus* strains, 2 *Bifidobacterium* strains, and a *Streptococcus* strain (60:25:15 ratio)	3 capsules per day (1 capsule thrice a day) for 4 months	In ASD children, Probiotic supplementation normalized Bacteroidetes/Firmicutes ratio ↓ *Desulfovibrio* spp. ↓ TNFα level in feces	[113]
Autistic children (4 to 10 years old)	*L. acidophilus* Rosell-11	5 × 10^9^ CFU per gram; twice a day for 2 months	↓ d-arabinitol, and d-arabinitol/l-arabinitol ratio in urine	[114]
ASD-Child (12 years old boy)	VSL#3 (a mixture of live cells of *Lactobacillus delbrueckii* subsp. *Bulgaricus*, *L. acidophilus, B. breve, B. longum, B. infantis*, *L. paracasei, L. plantarum, S. thermophiles*)	5 months of treatment period (4 weeks of initial treatment + 4 months of follow up treatment); 10 months of follow up period	↓ Severity of abdominal symptoms Improvement in autistic core symptoms	[115]
Autistic children (5 to 9 years old)	*B. longum*, *L. rhamnosus*, *L. acidophilus*	1 × 10^8^ CFU per gram; 5 g per day for 3 months	↑ Bifidobacteria and Lactobacilli level ↓ Severity of the ASD and GI symptoms	[116]
